# Longevity of mass-reared, irradiated and packed male *Anopheles arabiensis* and *Aedes aegypti* under simulated environmental field conditions

**DOI:** 10.1186/s13071-018-3191-z

**Published:** 2018-11-21

**Authors:** Nicole Jean Culbert, Hamidou Maiga, Nanwintoum Sévérin Bimbile Somda, Jeremie Roger Lionel Gilles, Jérémy Bouyer, Wadaka Mamai

**Affiliations:** 1 Insect Pest Control Laboratory, Joint FAO/IAEA Division of Nuclear Techniques in Food and Agriculture, Vienna, Austria; 2 Institute of Integrative Biology & the Centre for Genomic Research, University of Liverpool, Liverpool, Merseyside UK; 3 Institut de Recherche en Sciences de la Santé/Direction Régionale de l’Ouest (IRSS/DRO), Bobo-Dioulasso, Burkina Faso; 4 Institut de Recherche Agricole pour le Développement (IRAD), Yaoundé, Cameroon

**Keywords:** Sterile insect technique, Mosquito, Packing, Release time, Climatic conditions, Diptera

## Abstract

**Background:**

To ensure the success of a mosquito control programme that integrates the sterile insect technique (SIT), it is highly desirable to release sterile males with a maximal lifespan to increase release effectiveness. Understanding sterile male survival under field conditions is thus critical for determining the number of males to be released. Our study aimed to investigate the effect of mass rearing, irradiation, chilling, packing and release time on irradiated male mosquito longevity.

**Methods:**

*Anopheles arabiensis* and *Aedes aegypti* immature stages were mass-reared using a rack and tray system. Batches of 50 males irradiated at the pupal stage were immobilised, packed into canisters and chilled for 6 hours at 6 °C. Mosquitoes were then transferred either in the early morning or early evening into climate chambers set to simulate the weather conditions, typical of the beginning of the rainy season in Khartoum, Sudan and Juazeiro, Brazil for *An. arabiensis* and *Ae. aegypti*, respectively. The longevity of experimental males was assessed and compared to mass-reared control males subjected either to simulated field or laboratory conditions.

**Results:**

The combined irradiation, chilling and packing treatments significantly reduced the longevity of both *An. arabiensis* and *Ae. aegypti* under simulated field conditions (*P* < 0.001). However, packing alone did not significantly reduce longevity of *Ae. aegypti* (*P* = 0.38) but did in *An. arabiensis* (*P* < 0.001). Overall, the longevity of mass reared, irradiated and packed males was significantly reduced, with the median survival time (days) lower following an early morning introduction (4.62 ± 0.20) compared to an evening (7.34 ± 0.35) in *An. arabiensis* (*P* < 0.001). However, there was no significant difference in longevity between morning (9.07 ± 0.54) and evening (7.76 ± 0.50) in *Ae. aegypti* (*P* = 0.14)*.*

**Conclusions:**

Our study showed that sterile mass-reared males have a reduced lifespan in comparison to laboratory-maintained controls under simulated field conditions, and that *An. arabiensis* appeared to be more sensitive to the handling process and release time than *Ae. aegypti*. Longevity and release time are important parameters to be considered for a successful area-wide integrated vector control programme with a SIT component.

## Background

For several decades, the sterile insect technique (SIT) has been shown to be an efficacious and sustainable genetic approach with regard to the population management of several major pest insects, such as the New World screwworm *Cochliomyia hominivorax* [1], the tsetse fly *Glossina austensi* [2] and the Mediterranean fruit fly *Ceratitis capitata* [3], when deployed as part of an area-wide integrated pest management (AW-IPM) programme. Mosquitoes (Diptera: Culicidae) represent a serious threat worldwide for their vectorial capacity of major human disease pathogens. Several *Anopheles* and *Aedes* species are responsible for transmitting and spreading the most devastating disease pathogens including malaria, dengue, chikungunya, yellow fever, filariasis and the Zika virus. Over the last decade, substantial progress has been made regarding the development of the SIT package for mosquitoes including equipment and procedures [4]. The chikungunya and the unprecedented Zika virus outbreaks in the Americas in 2015 have further reignited interest in using the SIT to control mosquitoes.

There are many potential stressors a sterile male mosquito must endure before it is finally released into the field, including mass-rearing, sex-separation, irradiation, marking, handling, immobilisation and packing. It is assumed that each element imposes a slight cost on the quality of the insect itself. The irradiation process has been attributed to reduced male mating competitiveness in insects [5]. Thus, it is critical to determine the relative impact that each step has on insect quality to develop a standardised set of guidelines for each stage that imposes the least cost. However, there is little or no information regarding the post-pupal irradiation stages of mosquito SIT, such as handling, transport and release. Recently, optimal transportation conditions for sterile male *An. arabiensis* adults have been studied [6].

The release of sterile male mosquitoes within the framework of a large-scale programme may involve releasing the insects aerially. To achieve this, sterile male mosquitoes would have to be packed, stored and transported in large numbers. Thus, it is of interest to investigate the impact packing has on sterile male mosquito longevity and additionally the maximum density. Mosquitoes are produced in the laboratory under stable and favourable environmental conditions; however, they will be released into the field where environmental conditions undergo daily and seasonal variation. Thus, it is of concern how long these mass-reared sterile males will survive under challenging field conditions when released. Furthermore, it may be useful to determine if the time of day the release occurs has an impact upon the quality of the insect. Environmental conditions such as temperature and relative humidity (RH) can fluctuate drastically throughout the day; thus, preferred conditions need to be determined. Aerial releases will be most effective when carried out when the conditions are favourable, to optimise the insect’s survival and post-release performance. For example, aerial releases of sterile fruit flies are typically carried out early in the day; in Reynosa and Tijuana, both situated on the Mexican border, releases are carried out mid- and late morning, as is the case in the Los Angeles basin [7].

The work presented here aimed to estimate the survival of male mosquitoes when exposed to simulated field conditions and to determine the effect, if any, the process of packing has on sterile male longevity whilst undergoing chilling. Additionally, we investigated whether releasing irradiated males in the early morning or early evening was better by simulating natural environmental conditions for both *An. arabiensis* and *Ae. aegypti*. Lastly, we compared the longevity of irradiated males against unirradiated, mass-reared males that did not undergo chilling or packing, but were exposed to the simulated environmental conditions of Khartoum and Juazeiro or standard laboratory rearing conditions.

## Methods

### Source of mosquito colonies and mass rearing procedures

All experiments were performed using two established mosquito colonies, *An. arabiensis* (Dongola strain) and *Ae. aegypti* (Brazil strain), originating from the Northern State of Sudan (since 2005) and Juazeiro, Brazil (since 2012), respectively. Neither colony has been regenerated since the colonisation dates detailed above. They were maintained at the Insect Pest Control Laboratory (IPCL) of the joint Food and Agricultural Organisation/International Atomic Energy Agency (FAO/IAEA) Division of Nuclear Techniques and Agriculture, Seibersdorf, Austria, under controlled temperature, RH and light regimes (27 ± 1 °C, 70 ± 10% RH, 12:12 h light:dark (L:D) photoperiod with 1 h periods of simulated dawn and dusk). Eggs used for these experiments were generated following the *An. arabiensis* and *Ae. aegypti* mass-rearing procedures developed at the IPCL [8, 9]. Larvae were mass-reared in a large climate-controlled room (with an area of 88 m^2^) where temperature and humidity were maintained at 30 ± 1 °C, 70 ± 10% RH, respectively.

To mass rear *An. arabiensis*, 4 l of deionised water was added to each of the 50 larval mass rearing trays and placed within a mechanized stainless-steel rack developed at the IPCL [10]. Water was added 1 day before the addition of eggs to allow the water temperature to acclimatise to the ambient air temperature. Following the egg quantification method described in Maiga et al. [11], 4000 eggs were then added to each mass rearing tray, within a plastic ring floating on the water surface. Larvae were fed daily with 1% (wt/vol) IAEA diet developed and described at IAEA [12], using the feeding regime described in Soma et al. [13]. *Aedes aegypti* larvae were reared within mass rearing trays, with a larval density of approximately 18,000 first-instar larvae (L_1_) per tray containing 5 l of deionized water and fed with 7.5% IAEA diet (50 ml on day 1, 100 ml on day 2, 150 ml on day 3, 200 ml on day 4 and 50 ml from day 5 onwards) [14].

### Pupae collection and irradiation

Twenty-four hours after *An. arabiensis* pupae were first observed, the rack was tilted, and pupae separated from larvae by placing them in an Erlenmeyer flask containing dechlorinated water and swirling [15]. Male pupae were separated from females under a stereomicroscope by distinguishing differences in genitalia [15]. *Aedes aegypti* pupae were sexed mechanically by using a Fay-Morlan [16] glass sorter as redesigned by Focks (John W. Hock Co., Gainesville, FL, USA [17]) prior to further examination under a stereomicroscope, ensuring pure batches of males. To be consistent with ongoing field pilot trials by Member States, irradiation was carried out at the pupal stage. Twenty-four to 26-hour-old *An. arabiensis* pupae were exposed to 75 Gy, and 44–48-hour-old *Ae. aegypti* pupae were irradiated at 70 Gy in a self-contained ^60^Co Gamma Cell 220. Male pupae were irradiated in batches of 200 without water. The actual doses of irradiated pupae were quantified using Gafchromic MD film (International Specialty Products, NJ, USA). The actual doses received for *An. arabiensis* and *Ae. aegypti* were 86.5 ± 1 Gy and 77.5 ± 2 Gy, respectively.

### Setting up environmental field conditions inside climate chambers

**Fig. 1:**
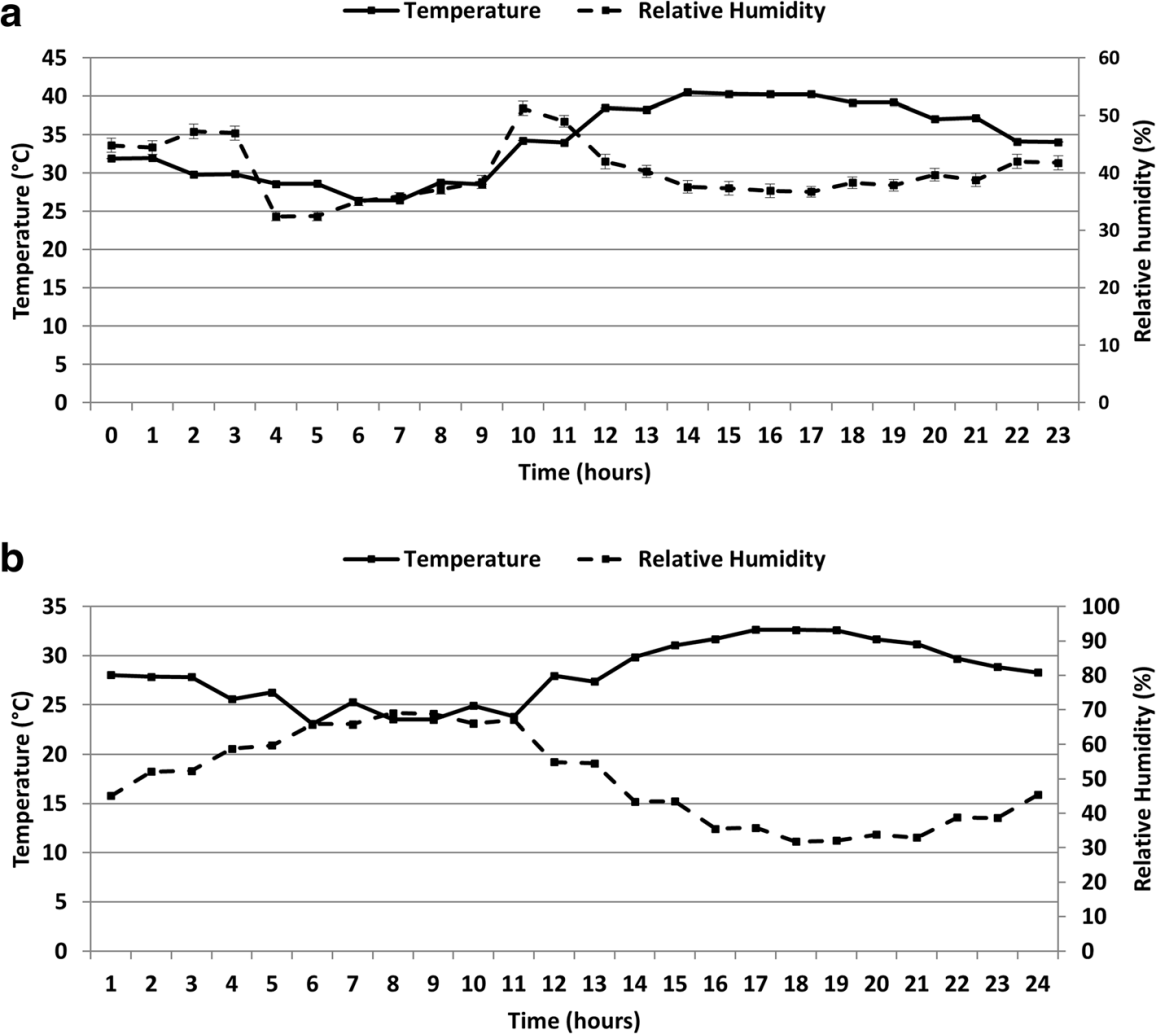
Mean (± SE) daily environmental conditions of temperature (solid line) and relative humidity (dashed line) recorded in the climate-controlled chambers simulating the natural conditions in Khartoum (Sudan) for *An. arabiensis* (**a**) and Juazeiro (Brazil) for *Ae. aegypti* (**b**)

Khartoum, Sudan and Juazeiro, Brazil environmental conditions were selected for the presence of these species and SIT pilot trials for *An. arabiensis* and *Ae. aegypti*, respectively. For *An. arabiensis*, the onset rainy season period was selected due to the fact that during the dry season, mosquito densities drop dramatically, and the mosquito population builds up gradually from the first rains toward the rainy season and in the northern part of Sudan, the seasonal larval population follows the rise and fall of the Nile River level [18]. We assumed therefore that this transition period (early rainy reason) could be the best period to start mosquito releases because the target mosquito population is already low and so that high ratios of sterile to wild insects would be easily obtained. A climate chamber (Sanyo MLR 315H, Osaka, Japan) was programmed to provide the temperature and RH on a typical April 17th, based on data obtained from a weather station at Khartoum International airport, Sudan, and averaged over 5 years. Twelve-step cycles were designed to reproduce the natural climatic variations monitored in the field. Experiments were conducted with a photoperiod of 12L:12D. The above process was repeated for *Ae. aegypti* with conditions set to simulate those of Juazeiro, Brazil, based on yearly hourly averages over 3 years. Data loggers (Onset Hobo data loggers, Bourne, MA, USA) were placed inside the chambers to monitor the temperature and humidity throughout the experiment. The actual data (averaged hourly records), presented in Fig. 1, simulated as closely as possible field data for Khartoum (Fig. 1a) and Juazeiro (Fig. 1b). Another chamber was set to 6 °C, 50% RH for the chilling process.

### Effect of packing on sterile male longevity

After the irradiation of pupae, sterile adults should be packed for transportation to the release area. Following irradiation, pupae were separated into batches of approximately 60 pupae (three replicates) and left to emerge in small Bugdorm cages (BugDorm, Taipei, Taiwan; 15 ×15 × 15 cm) with access to 5% and 10% sucrose solution for *An. arabiensis* and *Ae. aegypti*, respectively. On day 3 post-emergence, 3 cages containing 50 irradiated males were chilled at 4 °C for 5–10 min to immobilise the adults. After immobilisation, they were packed into plastic tubes (D × H: 1.5 × 4 cm) with an open end covered by a small square of mesh to allow ventilation and secured with an elastic band. Control males remained in their original Bugdorms and were not subject to packing. Both the experimental and control adults were placed inside a climate chamber set at 6 °C, 50% RH for a period of 6 h. After chilling, all cages were returned to laboratory conditions (27 ± 1 °C, 70 ± 10% RH) with experimental males removed from the packing tubes and returned to their original Bugdorm cages. Mortality checks were carried out daily in both control and experimental cages until no living adults remained.

### Assessing longevity of irradiated males under simulated field conditions and preferred time of day to release

Three batches of 50 sterile males, packed and chilled for 6 h were placed in the climate chamber at 6:00 h (treatment 1: morning) at the same time as the males which were not subjected to irradiation, packing or chilling (control 1: field conditions). Additionally, three batches of 50 control males which were not exposed to irradiation, packing or chilling were maintained at laboratory conditions (27 ± 1 °C, 70 ± 10% RH) (control 2: lab conditions). Three further batches of 50 sterile males were packed and chilled at 6 °C, 50 % RH for 6 h and then exposed to field conditions at 18:00 h (treatment 2: evening). Mortality was recorded daily until no adults remained. Unirradiated controls for the first 2 experiments were maintained under standard laboratory conditions.

### Statistical analysis

Graphics were produced and all statistical analyses were performed using Microsoft Excel 2013 (Microsoft®, Redmond, WA, USA) and GraphPad Prism v.5.0 (Windows, Graphpad Software, La Jolla California, USA; www.graphpad.com). The longevity of mosquitoes under various experimental conditions was analysed using Kaplan-Meier survival analyses. The log-rank (Mantel-Cox) test was used to compare the level of survival between different treatments and controls. To counteract the problem of multiple comparisons the Bonferroni correction method was applied for each pair of groups.

## Results

### Effect of packing on male longevity of *Anopheles arabiensis* and *Aedes aegypti*

**Fig. 2:**
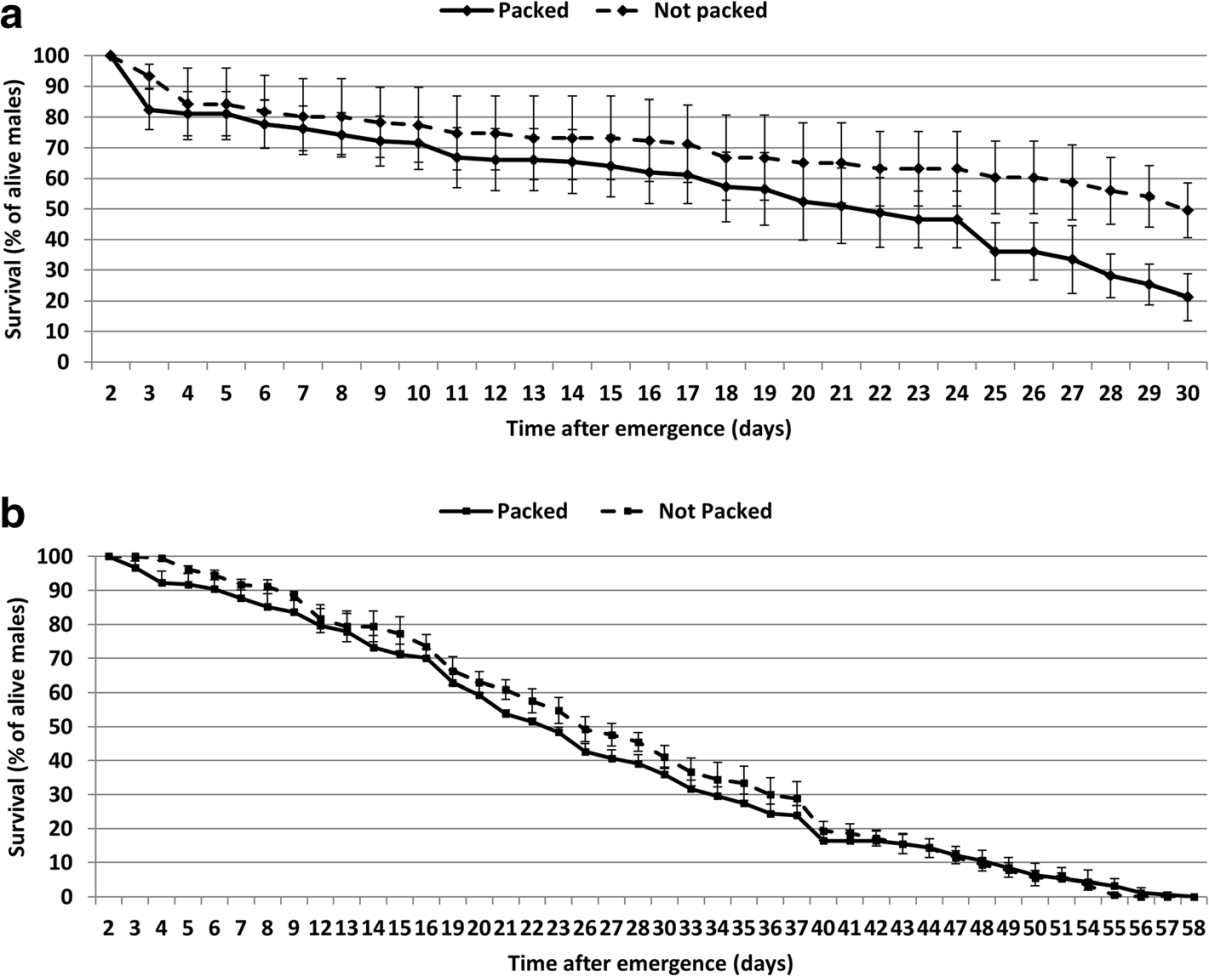
Mean (± standard error, SE) longevity of male *Anopheles arabiensis* (**a**) and male *Aedes aegypti* (**b**) recorded under packed (solid line) and unpacked (dashed line) conditions

The longevity of 50 sterile males packed in a small tube and chilled at 6 °C for 6 h was compared to 50 sterile males chilled at 6 °C for 6 h in a small Bugdorm cage. The longevity was followed in laboratory conditions and the survival curve is presented in Fig. 2. Statistical tests between all treatments were summarized in Table 1. The analyses showed that the packing treatment significantly reduced the longevity of *An. arabiensis* males (Fig. 2a, Table 1, Log-rank (Mantel-Cox) test *χ*^2^ = 18.15, *df* = 1, *P* < 0.001) and did not affect that of *Ae. aegypti* males (Fig. 2b, Table 1, Log-rank (Mantel-Cox) test *χ*^2^ = 0.76, *df* = 1, *P* = 0.38).

**Table 1:** Results of log-rank (Mantel-cox) test analysis for the effect of packing, environmental treatments and preferred time of day to release on the longevity of *Anopheles arabiensis* and *Aedes aegypti* males

	Treatments for comparison	*χ* ^*2*^	*df*	*P*
*An. arabiensis*	Packed × unpacked	18.15	1	<0.001
	Treatment 1 (Morning) × Treatment 2 (Evening)	41.09	1	<0.001
	Treatment 1 (Morning) × Control 1 (field conditions)	80.45	1	<0.001
	Treatment 1 (Morning) × Control 2 (lab conditions)	331.00	1	<0.001
	Treatment 2 (Evening) × Control 1(field conditions)	15.60	1	<0.001
	Treatment 2 (Evening) × Control 2 (lab conditions)	91.45	1	<0.001
	Control 1 (field conditions) × Control 2 (lab conditions)	274.30	1	<0.001
*Ae. aegypti*	Packed × unpacked	0.76	1	0.38
	Treatment 1 (Morning) × Treatment 2 (Evening)	2.21	1	0.14
	Treatment 1 (Morning) × Control 1 (field conditions)	149.70	1	<0.001
	Treatment 1 (Morning) × Control 2 (lab conditions)	363.60	1	<0.001
	Treatment 2 (Evening) × Control 1(field conditions)	176.20	1	<0.001
	Treatment 2 (Evening) × Control 2 (lab conditions)	409.70	1	<0.001
	Control 1 (field conditions) × Control 2 (lab conditions)	124.00	1	<0.001

### Longevity of *An. arabiensis* and *Ae. aegypti* males under different environmental treatments and time of day to release

**Fig. 3:**
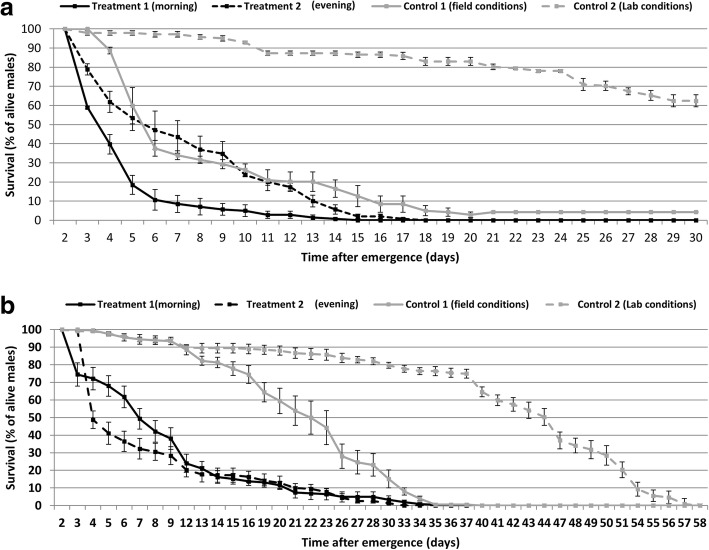
Mean (± standard error, SE) longevity of male *Anopheles arabiensis* (**a**) and male *Aedes aegypti* (**b**) under different treatments. Treatment 1: mass-rearing + irradiation + packing + field conditions + introduced in the climate chamber at 6:00 h. Treatment 2: mass-rearing + irradiation + packing + field conditions + introduced inside the climatic chamber at 18:00 h. Control 1: mass-rearing + field conditions + introduced inside the climatic chamber at 6:00 h. Control 2: mass-rearing + laboratory conditions + introduced in the lab at 6:00 h

When exposed to simulated field conditions, the combination of irradiation, chilling and packing (treatment 1 *vs* control 1 and treatment 2 *vs* control 1) significantly reduced the longevity for *An. arabiensis* (Fig. 3a, Table 1, Log-rank (Mantel-Cox) test *χ*^2^ = 80.45, *df* = 1, *P* < 0.001 and *χ*^2^ = 15.60, *df* = 1, *P* < 0.001 for treatment 1 *vs* control 1 and treatment 2 *vs* control 1, respectively) and *Ae. aegypti* (Fig. 3b, Table 1, Log-rank (Mantel-Cox) test *χ*^2^ = 149.7, *df* = 1, *P* < 0.001 and *χ*^2^ = 176.2, *df* = 1, *P* < 0.001 for treatment1 *vs* control 1 and treatment 2 *vs* control 1). In addition, the combination of irradiation, chilling, packing and laboratory conditions (treatment 1 *vs* control 2) significantly reduced male longevity for *An. arabiensis* (Fig. 3a, *χ*^2^ = 331.0, *df* = 1, *P* < 0.001) and *Ae. aegypti* (Fig. 3b, *χ*^2^ = 363.6, *df* = 1, *P* < 0.001).

The exposure of male mosquitoes to simulated field conditions (control 1) significantly reduced longevity compared to those maintained under laboratory conditions (control 2) for *An. arabiensis* (Fig. 3, *χ*^2^ = 274.3, *df* = 1, *P* < 0.001) and *Ae. aegypti* (Fig. 3b, *χ*^2^ = 124.0, *df* = 1, *P* < 0.001).

For *Ae. aegypti*, there was not significant effect on longevity (Fig. 3b, *χ*^2^ = 2.209, *df* = 1, *P* = 0.1372) for males introduced inside the climatic chamber (field conditions) in the morning (6:00 h) or in the evening (18:00 h). The median survival time was 9.07 ± 0.54 and 7.76 ± 0.50 days for morning and evening, respectively (Table 2). Conversely, for *An. arabiensis*, males introduced inside the climatic chamber in the evening (18:00 h) had a higher longevity than those introduced in the morning (6:00 h) (Fig. 3a, Log-rank (Mantel-Cox) test *χ*^2^ = 41.09, *df* = 1, *P* < 0.001). The median survival time was 4.62 ± 0.20 days for the morning and 7.34 ± 0.35 days for the evening (Table 2).

**Table 2:** Mean (± SE) longevity (days) of *Anopheles arabiensis* and *Aedes aegypti* males exposed to different environmental treatments For each treatment, *n* = 3 replicates, 50 mosquitoes/replicate

Species	Treatment 1 (morning)^a^	Treatment 2 (evening)^b^	Control 1 (field conditions)^c^	Control 2 (lab conditions)^d^
*An. arabiensis*	4.62 ± 0.20	7.34 ± 0.35	8.33 ± 0.42	19.09 ± 1.07
*Ae. aegypti*	9.07 ± 0.54	7.76 ± 0.50	21.28 ± 0.56	39.94 ± 0.98

^a^Treatment 1: mass-rearing + irradiation + packing + field conditions + introduced in the climate chamber at 6:00 h

^b^Treatment 2: mass-rearing + irradiation + packing + field conditions + introduced inside the climatic chamber at 18:00 h

^c^Control 1: mass-rearing + field conditions + introduced inside the climatic chamber at 6:00 h

^d^Control 2: mass-rearing + laboratory conditions + introduced at the lab at 6:00 h

## Discussion

Understanding sterile male longevity is of utmost importance for the effective implementation of SIT technology. Our research aimed to investigate the longevity of sterile male mosquitoes when exposed to simulated field conditions. Additionally, we aimed to determine whether there is an effect of the process of packing on sterile male mosquito longevity. The impact of packing sterile males, such as would be performed prior to transporting adults from a rearing facility to a release site was explored. Additionally, we simulated environmental conditions for morning and evening releases for both *An. arabiensis* and *Ae. aegypti* to determine whether time of day had any impact upon subsequent longevity.

Packing was found to significantly decrease the longevity of *An. arabiensis.* This result is inconsistent with what was noted when comparing the longevity of compacted *vs* non-compacted *An. arabiensis* in an earlier publication, where no significant decrease was observed [6]. The different methodology between the experiments within this study and that of our earlier publication may indeed have contributed to the different results. For example, in our previous study, male *An. arabiensis* were not subject to irradiation. Perhaps this is why packing did not significantly decrease survival between packed and unpacked experimental males, but in comparison to control males, both experimental treatment groups (packed and non-packed) did display a significantly lower longevity [6]. In the packing experiment detailed within this manuscript, males were subject to irradiation. Therefore, it may be a synergetic effect of irradiation, packing and chilling which caused a significant decrease in longevity for *An. arabiensis* in our study. This synergetic effect has been shown in other species used within programmes with a sterile insect component. Sterile male fruit flies (*Ceratitis capitata*) were observed to exhibit significantly reduced flight ability and mating competitiveness when chilled in crowded conditions. However, independently, chilling or crowding did not cause significant decreases in either parameter [6, 19]. Interestingly, the same result was not observed for *Ae. aegypti*. *Aedes aegypti* and *Ae. albopictus* mosquitoes appear to be less susceptible to chilling and compaction as compared to *Anopheles* (Culbert, unpublished data). We suspect this divergent response between species may reflect their different levels of tolerance to stressors. However, it cannot be ruled out that other factors, such as long-term colonisation in *An. arabiensis* (13 years, without regeneration), might be involved in causing the fragility observed during packing.

*Anopheles arabiensis* exposed to simulated field conditions when released in the evening had a significantly higher survival rate compared to those released at early morning. In the evening, conditions were much warmer (around 39 °C) than that in the morning (26 °C). However, temperatures increase in the morning (from 26 °C to 45.5 °C) while in the evening temperatures decrease (from 39 °C to 34 °C). *Anopheles arabiensis* exposed to high and decreasing temperatures seem to adapt much better than those exposed to low and increasing temperatures. It has been demonstrated that a brief exposure to extreme heat or cold often elicits physiological responses such as heat shock proteins that improve an organism’s thermal tolerance [20]. *Anopheles arabiensis* is well known to favour hot, dry conditions in the wild [21], most notably in Sudan [22], the origin of our laboratory strain. This may have contributed to the higher survival observed in males which underwent a simulated evening release but conflicts with the literature, which states insects generally lose their thermal tolerance upon domestication [23]. There is considerable variation regarding *Aedes* survival in the field, due to the limited temperature ranges at which field studies are conducted in addition to the relatively small sample sizes used in mark release recapture (MRR) studies [24]. In *Ae. aegypti*, we found no significant difference in longevity between an early morning and an evening release. This may be because the shift in temperature between morning and evening was not as great as that which *An. arabiensis* were subjected to, with the fluctuation range closer to their normal rearing conditions within the laboratory.

Sterile male insects have one purpose, to mate with wild females and thus induce sterility within the target population. It is critical that sterile insects survive as long as possible in the field to ensure the success of a SIT programme. If sterile males are of poor quality and exhibit a reduced longevity, the frequency of releases coupled with the number of insects required for each release will have to be increased in order to preserve the overflooding ratio [25], which will increase costs. Mass-rearing, irradiation, handling and release methods can all contribute to a reduced lifespan in sterile insects. Often, longevity studies are conducted within the laboratory and may not be an accurate reflection of actual field survival; for example, due to the controlled climatic conditions and the absence of predation. This may explain why our results, when conducted to more accurately reflect a field setting, differ from what has previously been observed within our laboratory studies. *Anopheles* males have been shown to survive on average for 20 days within a laboratory setting, whilst wild types display a much shorter lifespan averaging only 5–10 days [26]. In *Aedes* species, wild male longevity is less documented but does seem to be dependent on season, with *Ae. aegypti* populations in Vietnam found to exhibit a much higher survival in either cool or hot dry seasons when compared to the cool and wet season [27].

## Conclusions

The results of this study highlight the fact that each step before release, such as the mass-rearing process, irradiation, handling and transport, can cause a cumulative detrimental effect on the longevity of sterile mosquitoes and perhaps their overall quality. This is further emphasised when conditions are set to simulate field conditions, as opposed to a controlled laboratory setting. It would be of interest to conduct quality control tests, such as investigating flight ability and or mating competitiveness experiments, to ascertain if these parameters are impaired too. Understanding which treatments impact sterile male quality most and rectifying those parameters will ultimately lead to a higher quality of insect and a more successful SIT programme.

## Abbreviations

FAO: Food and Agricultural Organization
IAEA: International Atomic Energy Agency
IPCL: Insect Pest Control Laboratory
L:D: light:dark
RH: Relative humidity
SIT: Sterile insect technique
